# Factors Influencing Development of Professional Values Among Nursing Students and Instructors: A Systematic Review

**DOI:** 10.5539/gjhs.v7n2p284

**Published:** 2014-11-16

**Authors:** Akram Parandeh, Morteza Khaghanizade, Eesa Mohammadi, Jamileh Mokhtari Nouri

**Affiliations:** 1 Research Center of Medicine and Religion, Baqiyatallah University of Medical Sciences, Tehran, Iran; 2 School of Nursing, Baqiyatallah University of Medical sciences, Tehran, Iran; 3 Department of Nursing, Medical Sciences Faculty, Tarbiat Modares University, Tehran, Iran

**Keywords:** ethic, nursing education, nursing students, profession development, professional values, systematic review, values

## Abstract

**Introduction:**

Professional values are standards of behavior for performance that provide a framework for appraising beliefs and attitudes that influence behavior. Development of professional values has been a continuous and long process and it is influenced by different factors. The aim of this study is “assessing different factors influencing development of professional values among nursing students and instructors”.

**Method:**

In this systematic review, a broad research was performed to find articles from Persian and English databases: pub Med, Pro quest, Elsevier, SID, Google scholar, Ovid and Iran Doc; nursing student, instructors, ethics, professional value, ethical value and educators were used as the key words. Among 3205 achieved articles, by eliminating repeated ones, 22 articles were assessed during the period 1995–2013. Data achieved from the articles were summarized, categorized and analyzed based on the research question.

**Results:**

In this study “education and achieving professional experiences”, “Students and instructors’ perspectives on professional values”, “the role of culture in considering and developing professional special values” and “the effect of learners’ individual characteristics” were extracted as the four main themes.

**Conclusion:**

Considering the effect of educational, cultural and individual factors in developing nurses’ professional values; it is recommended to the educational and health centers to consider value-based cares in clinical environments for the patients in addition to considering the content of educational programs based on ethical values in the students’ curriculum.

## 1. Introduction

Nurses as a member of the health system and health service providers are responsible for providing care to the patients based on ethical issues (Johnstone, 2004). Nowadays, in the changing world, nurses need ethical knowledge for appropriate performance, management of the situations and providing safe and appropriate legal and ethical services (Chitty & Black, 2007). Professional values are the performance standards that are accepted by the professional and specialist group (Schank & Weis, 2001), these values are the base of nursing performance, the director of the nurses’ interaction with the patients, colleagues and other professionals and the public (Leners, Roehrs, & Piccone, 2006) and also as a guideline for ethical behavior to provide security and humanitarian care (Lawler, 2008). Actually, values are goals and beliefs that create behaviors and they are a basis for decision making and practice (Chitty & Black, 2007; Martin, Yarbrough, & Alfred, 2003). Personal values will be developed through being influenced by family, culture, society, environment, religious belief and ethnicity (Blais, 2010). Acquisition of these values is a gradual and evolutionary process which happens throughout people’s life (Leners et al., 2006). Professional values are in relationship with individual believes and they are mostly rooted in personal values and it is as something that is considered good or appropriate for the members of every profession (Blais, 2010). The acquisition and internalizing professional values are necessary in care setting for professional development (Horton, Tschudin, & Forget, 2007) and providing a common framework for meeting professional expectations and standards besides increasing ethical dilemmas (Irving & Snider, 2002). Therefore, developing nursing professional values is important because of promotion of care quality, increase of patient’s understanding, increase of job satisfaction and retention of nursing staff (Horton et al., 2007); it also helps the professional socialization process (Blais, 2010).Students, educators, college, clinical and educational experiences, lectures, the experience of taking care of a patient and also individual values are the among significant components of learning and developing professional values (Clark, 2009). Schank and Weis (2001) believes that; achievement of educational experiences is very effective in developing students’ professional values (Schank & Weis, 2001). Evaluating professional values in the students’ perspective can establish helpful information for providing more effective strategies to integrate and utilize professional values in ethical performance and clinical learning (Lui et al., 2008). Leners et al. (2006) also states that; ethical performance of the nursing instructors significantly influences the formation and growth of the students’ professional values during educational programs (Leners et al., 2006). So considering the lack of systematic studies in this regard and the importance of understanding and explaining professional values in the students and instructors’ perspective and its effect on the growth of the profession; the present study is conducted for “assessing factors influencing development of professional values among instructors and students based on a systematic review”.

## 2. Methods

The present systematic review is conducted for answering the research question which is: “how the factors influencing development of professional values among nursing students and instructors are described and explained in the nursing literature?”

**Figure 1:**
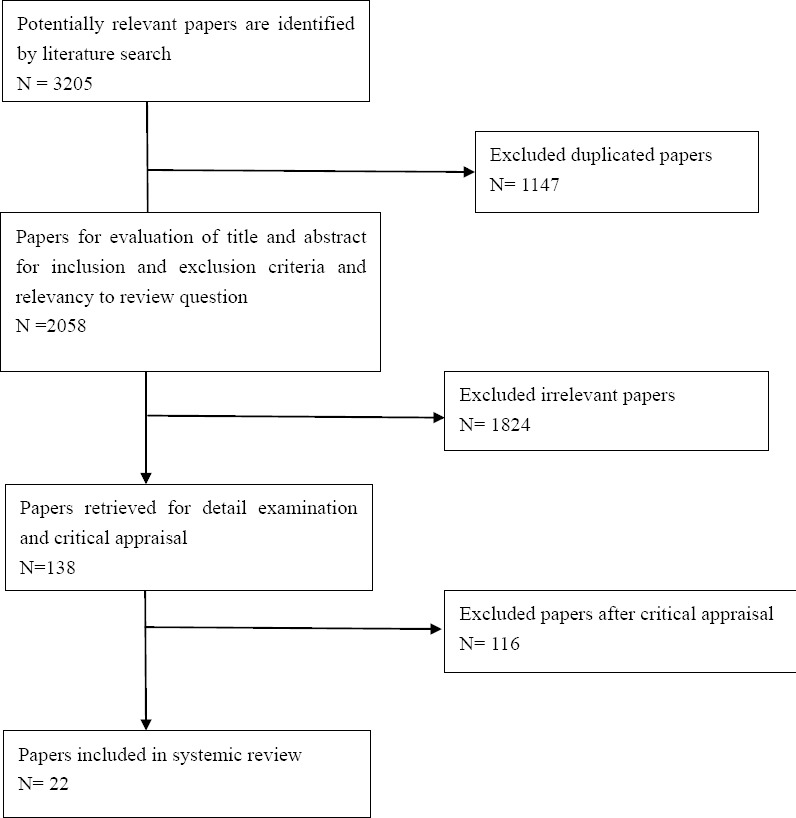
Process of searching and reducing records for the systematic review

Inclusion and exclusion criteria: all the published articles and thesis about nurses’ professional values and nursing students in English and Persian language in the period 1995-2013, original articles with qualitative and quantitative approaches and review literature and mixed- method study. Exclusion criteria included: anonymous articles, a review chapter of a book, recommendations, letter to the editor and historical articles. In order to achieve articles, a broad research was done in SID, pub Med, Pro quest, Elsevier, Google Scholar, Ovid and Iran Doc databases. In a preliminary research, at first, separated key words and then combination of key words were used by using AND/OR for combining the words, the used key words included: nursing student, nursing instructor, ethics, professional value, ethical value and educators. Results achieved from the review were summarized in several stages including: the first stage in which 3205 topics and abstract of the articles were identified in all the informational databases by using combined and main key words and they were imported into the Endnote X4 software (Thomson Reuters, New York) and the record were sorted. After the removal of duplicates (1147 articles), a number of the articles were decreased to 2058 topics. In the second stage, these articles were assessed based on inclusion and exclusion criteria and related to the research question which was a description of professional values focusing on students and instructors. Then 1824 unrelated studies were extracted from the study. In the third stage, papers were retrieved for detail examination and critical appraisal; numbers of the articles were decreased to 138 ones. In the fourth (final) stage, Twenty-two articles met the criteria of the systematic review. The process of reducing and evaluating the records is shown in Figure 1. In the present study, most of the articles are described; a qualitative study with Q methodology approach (Akhtar-Danesh et al., 2013), a mixed -method study (qualitative and quantitative) (Rognstad, Nortvedt, & Aasland, 2004) and three studies of theoretical design (Fahrenwald et al., 2005; Shaw, & Degazon, 2008; Weis, & Schank, 2002) were used for analysis. General characteristics of the studies are listed in Table 1. For data extraction and synthesis, articles had been read by one of the reviewers carefully; the most important points of the articles were summarized along with the aim of the research and these points were extracted and sorted by narrative summary. Finally, findings were reported based on thematic content analysis in the form of related themes. For increasing the study accuracy, extracted data were controlled and reviewed through reviewing the process by three other researchers of the research team.

**Table 1:** General characteristics of the review studies

Focus of the studies	Author’s/years	Samples/design of the study	Conceptual framework/tools	Data collection	Important findings
education and achieving professional experiences,	Fahrenwald (2005) Shaw (2008) Bang et al. (2011) Lin (2010), Rassin (2010), Schank and Weis (2001), Leners et al. (2006), Weis and Schank (2002),	991 nursing students 22 nurses Quantitative and descriptive cross-sectional and longitudinal cross-sectional design, Theoretical study design,	Schank and Weis 44-questionnaire, Israel ethical codes questionnaire (1996 and 2004) Korea professional values questionnaire ANA, AACN and ICN conceptual framework	Questionnaire	Teaching professional values influence development of students’ professional values positively. Professional values are related to students’ interest to the profession. Nursing instructors’ can influence the students’ professional value development by performing role modeling.

Students and instructors’ perspectives on professional values,	Par van et al. (2012), Schank and Weis (2001), Leners et al. (2006), Lin (2010), Bang et al. (2011), Lui (2008), High (2007), Eddy et al. (1994), Akhtar-Danesh (2013), Rognstad et al. (2004), Clark (2009), Kalb (2007),	32303 nursing students, 396 nursing instructors, Quantitative study with descriptive, longitudinal, cross-sectional design, Qualitative study with Q methodology, Mixed- method (qualitative and quantitative),	Schank and Weis 44-questions questionnaire, Norway professional values questionnaire, Honk-Kong professional values questionnaire, ANA, AACN and ICN conceptual framework,	Questionnaire Interview	Students’ perspective regarding professional values has been influenced by their approaches towards professional values. Students often believe in, care dimension of professional values, while instructors consider values such as; human dignity in high priority. There is an agreement regarding the nurse-patient relationship in both groups as an important professional value.

The role of culture in considering and developing professional special values,	Konishi et al. (2009), Alfred et al. (2011,) Martin et al. (2003), Schank and Weis (2000), Wang (1994), Weis and Schank (1997),	1842 nursing students, 30 instructors, Quantitative study with cross-sectional descriptive design, Qualitative study,	Schank and Weis 44questions questionnaire, professional value questionnaire Rokeach China (RVS), ANA and AACN conceptual framework	Questionnaire Interview	Although ethics and professional values are global, culture has formed them and it counts as a very important factor in choosing the kind of professional value among students, nurses and instructors.
The effect of learners’ individual characteristics	Martin (2003), Hayes (2006), Par van et al. (2012), Eddy et al. (1994), Bang (2011), Lin (2010), Loui (2008),	55340 nursing students, 350 instructors, Quantitative study with descriptive qualitative design	44questions questionnaire Schank and Weis, ANA and AACN conceptual framework,	Questionnaire	Gender as one of the individual characteristics influence type of professional values, so that in most of the studies, professional values scores of females are reported higher than males.

## 3. Results

Based on the systematic review regarding factors influencing development of professional values of the nursing students and instructors, results achieved from the study can be explained in the form of the following themes:

·***Education and achieving professional experiences***

Results of the studies showed that; education and achieving learning experiences influence growth and development of professional values positively (Bang et al., 2011; Leners et al., 2006; Lin, Wang, Yarbrough, Alfred, & Martin, 2010; Rassin, 2010; Schank, & Weis, 2001; Weis, & Schank, 1997, 2002). Findings in addition to emphasis on paying attention to value-based integrated education in the entire curriculum stated that; education cause practical, conceptual and ethical learning. So that purposeful integration of value-based education in the nursing curriculum causes the application of professional values such as; human dignity, altruism and social justice in clinical work and it is going to be as a legacy of caring behavior and a strong positive point for the nurses’ work in the future (Fahrenwald et al., 2005). Educating professional values in nursing curriculum by emphasizing on three cognitive, mental and emotional domains facilitates and promotes professional and acculturation development process. So that creating and developing creative methods for educating professional important values is as a bridge for usual understanding and obligation among lots of differences in art and science of nursing and causes provision of nursing cares for individuals, family and society according to the culture (Shaw & Degazon, 2008; Weis, & Schank, 2002). The results of the studies indicated that; students’ achievement of learning experiences increases the total score of professional values and has a positive effect on professional values from the time of entering to exiting from nursing education course (Bang et al., 2011; Leners et al., 2006; Lin et al., 2010). Studies showed that; there is a significant difference between achieving theoretical knowledge of a value and applying that, because development of professional values occurs in a long continuum that is started from educational environments and is developed gradually in supplying clinical activities and achieving work experiences (Rassin, 2010; Schank, & Weis, 2001). Considering the results; although education influences professional values positively, students do not have adequate basic knowledge of professional values and they had different perspectives in this regard (Fahrenwald et al., 2005; Lin et al., 2010; Shaw, & Degazon, 2008).

·*Students and instructors’ perspectives on professional values*

Students and instructors’ perspective and understanding about professional values have been explained in 11 studies of 22 studies (Akhtar-Danesh et al., 2013; Bang et al., 2011; Clark, 2009; Eddy, Elfrink, Weis, & Schank, 1994; Haigh, & Johnson, 2007; Leners et al., 2006; Lin et al., 2010; Lui et al., 2008; Parvan, Zamanzadeh, & Hosseini, 2012; Rognstad et al., 2004; Schank, & Weis, 2001). Students’ perspectives on professional values have influenced decision making and the way of taking care for the patient. Also, instructors can influence internalization of professional values in the students’ performance by considering the role of students’ practical and behavioral patterns. The findings of the studies showed that; students consider trust and care as the important dimensions of their professional values. Also, most of the students do not have adequate knowledge about the importance of any dimensions and value components related to non-clinical duties (Parvan et al., 2012). Studies showed that; values such as respecting human, privacy, confidentiality of patients, support and care with high quality that all are related to the care dimension are very valuable for the students and nurses (Leners et al., 2006; Schank, & Weis, 2001). Students have different perspectives on professional values during their educational courses, so that professional values at the time of students’ entry to the university were mostly about clinical care aspects and the features of the patient-nurse relationship and at the time of graduation, professional values were mostly about aspects of patient advocacy. Also students’ perceive and perspective on nursing was considered as a caring profession (Lin et al., 2010). Findings showed that; providing secure and appropriate care for the clients is stated as the most important facet of professional conducts by the students and nurses (Bang et al., 2011; Clark, 2009; Lui et al., 2008). Results of an international study about nursing instructors of 19 different countries showed that; nursing instructors reported honesty and intellectualism professional value very important with a high percentage and considered the altruism value in the clinical environments important too (Haigh & Johnson, 2007). Regarding comparison of students and instructors’ perspective on professional values, results indicated that scores of instructors’ professional values were higher than the students. Instructors’ important professional values, including equality, human dignity and freedom were as the important values of the students too (Eddy et al., 1994). Study of Akhtar-Danesh et al. (2013) regarding perceptions of professionalism among nursing faculty and nursing students showed that; humanism is one of the important concepts of professionalism and professional values. So that humanism indicates professional values approach, including: “respecting human dignity, personal integrity, protecting patient’s privacy, protecting patients from harm, responsibility, paying attention to the personal beliefs and values and also standards and policies” (Akhtar-Danesh et al., 2013). Studies have shown; that most of the students chose the nursing profession because of their desire for communicating human being and helping them, therefore helping human means appreciation and altruism in their perspective. They consider altruism as helping to another person or providing care for another person because he/she is asking for help (other-concern) and they stated appreciation value as a positive feedback given by one who received help (self-concern)(Rognstad et al., 2004). Clark (2009) revealed that; nurses and students perceive patient care values to be more important than nursing values related to promoting the nursing, being a member of a profession or participating in research (Clark, 2009). In the study of Eddy (1994), esthetic professional value was very important in the students’ perspective, because this approach is due to their type by looking at the ideal work environments. Most of the students and nurses after entering into practice liked to learn most of the available routines of the unit and perform patients’ clinical care skills, sometimes they did not consider a professional value such as; patient’s refusal of treatment as an ethical subject and most of them were unfamiliar with patients’ rights (Eddy et al., 1994).

·***Role of culture in considering and developing professional special values:***

Culture was one of the very important factors in developing individuals’ professional values in the studies (Alfred, 2011; Konishi, Yahiro, Nakajima, & Ono, 2009; Martin et al., 2003; Wang, Rao, & D’Auria, 1994; Weis, & Schank, 1997). The most important professional value and virtue in Japanese culture is reported politeness and respecting others in the results of the conducted study among the nurses (Konishi et al., 2009). Results of the comparative study regarding professional values among nursing students of Taiwan and America showed that; although professional values scores between the two groups were high and there was not any remarkable significant difference, the difference of some of professional values components between the two groups were due to culture(Alfred, 2011). In the study by Martin et.al (2003) about congruence of professional values between the two groups of the students, results indicated different professional values among different ethnicity with different cultural perspectives, so that responsibility score in three important professional values of respecting human dignity, safeguarding the client and the public, participation and cooperation in meeting health needs of the community in Asian/Pacific race was less than others(Martin et al., 2003). But findings of the study of Weis and Schank (1997) showed that; there was no difference in the total score of professional values among nursing students of America and England, so that similarities regarding professional values are more than the differences between the two groups and the differences may have resulted from cultural Factors (Weis & Schank, 1997). Some values such as: “being honest, ambitious and responsible” have always been the most important instrumental values for America and “happiness, politeness and being independent” have been among the most important values in China (Wang et al., 1994). Also, considering differences of professional values among different cultures, Alfred et al. (2011) compared the professional values of nursing students in Taiwan and the U.S.; Taiwanese students named some of caring factor statements as the most important statements including: “Maintaining patient’s confidentiality”, “Safeguarding patient’s right to privacy” and “Practicing principles of fidelity and respecting human”. Furthermore, the most important statements of the American students’ perspectives were “Acting as patient advocate”, “Maintaining patient’s confidentiality” and “Protecting moral and legal rights of patients” (Alfred, 2011). Also study of Schank and Weis (2000) was about professional values difference between American and British nursing instructors; it was related to social aspects by focusing on nurses’ responsibility towards society and profession. Actually, these differences indicated differences in cultural, educational and health care systems (Schank & Weis, 2000).

·***The effect of learners’ demographic characteristics:***

A review on the studies showed that; many individual factors such as: age, gender and educational level influence professional values (Bang et al., 2011; Eddy et al., 1994; Hayes, 2006; Lin et al., 2010; Lui et al., 2008; Martin et al., 2003; Parvan et al., 2012). There is a significant relationship between educational level and professional values scores in some studies. So that in some studies, the scores average in female students is higher than males (Bang et al., 2011; Hayes, 2006; Martin et al., 2003) and in some others, it is vice versa; scores of male students are higher than females (Lin et al., 2010; Lui et al., 2008), also in some studies, there was no significant relationship between gender and professional values (Eddy et al., 1994; Parvan et al., 2012). Results of the study of Martin et al (2003) showed that; respecting human dignity, safeguarding the client and public, and participating to meet public health needs were as the different values among both males and females, also the value of a patient’s privacy, assuming responsibility and accountability, effort in making an informed judgment, application and promotion of nursing standards and collaboration with others in students of associate degree were higher than students of bachelor degree of the same year in nursing students (Martin et al., 2003). In the study of Bang et al. (2011), there was a relationship between professional values with the level of students’ interest and tend to the profession and talent (Bang et al., 2011).

## 4. Discussion

Results achieved from the systematic review showed that; education and achieving professional experiences, students and instructors’ perspective on professional values, culture and learners’ individual characteristics have been as the four main themes of the important factors in developing professional values. *Education and achieving professional experiences* have an important role in the growth and development of professional values. Studies have shown that; education does make a difference in the professional values formation and educators are influential in instillation of the professional values essential to professional development (Weis & Schank, 2002). Teaching and role modeling of the educators are always important in strengthening students’ professional values even with the absence of specific ethics course in the curriculum (Elfrink & Lutz, 1991). Eddy (1994) quoted from Banner (1985) that; instructors can prepare students for moral decision making and professional value growth through helping in socialization process of the students (Eddy et al., 1994). Elfrink and Lutz (1991) also state that; in most of the cases values and ethics issues were not formally a part of the curriculums but were educated “incidentally” through unplanned classroom and clinical discussions (Elfrink & Lutz, 1991). Weis and Schank (2002) state that; professional values are going to be developed and progressed through education, and they are going to be facilitated and developed through instructors’ role modeling behaviors (Weis & Schank, 2002). Therefore the students’ professional values are changed by entering the profession during educational processes and by achieving clinical experiences based on professional norms and standards (*American Association of Colleges of Nursing The essentials of baccalaureate education for professional nursing practice*, 1998). Regarding *students and instructors’ perspective*, findings state shortage of students’ knowledge and awareness of their professional values at the time of education, students and instructors had a different approach towards the type of professional values. In this regard, Fahrenwald et al. (2005) emphasized that; for all-aspect knowledge and promotion of the nursing professional values, planning and applying appropriate educational methods with more exact content regarding professional values is an essential principle (Fahrenwald et al., 2005). Passing time, in addition to influencing the development of professional values has an important role in the type of students and instructors’ perspective. So that based on the students and instructors’ perspective, the patient-nurse relationship is a very important value and according to the both groups’ perspective, this value is in high priority. One of the interesting findings in the systematic review was that students often pay more attention to the care dimension of professional values by the entering nursing profession; it is while instructors mostly pay attention to some values such as: respecting human dignity, honesty and altruism. Although, the basis of the nursing profession is taking care of the patient, according to a professional behavior, it is the main axis of ethical and professional codes of the nurses (*American Nurses Association Code of ethics for nurses with interpretive statements*, 2001). In concurrence with this finding, Fahrenwald (2005) states that; nursing care is going to be provided in its perfect form when nurses can integrate important professional values with care (Fahrenwald et al., 2005). Another significant finding of reviewing these studies was the importance of respecting human dignity in both students and instructors’ perspective, so that students despite having different perspectives on human dignity stated it as one of their important professional values (Kalb & O’Conner-Von, 2007; Akhtar-Danesh et al., 2013; Eddy et al., 1994; Rassin, 2010). Nowadays, paying attention to patients’ human dignity is the important issue of the health care environments. Based on Code of Ethics with Interpretive Statements, human dignity is the first and the most important ethical principle in nursing (*American Nurses Association Code of ethics for nurses with interpretive statements*, 2001). Nursing art is based on the framework of care and respecting human dignity. Therefore, all the professional values, especially human dignity should be considered in the programs of nursing education. The results of the studies showed that; professional values are considered by many *cultures*. Culture is defined as a set of values, beliefs, and habits learned in social, that shape the world of ideas, perceptions, decisions, and how people performance. Professional caregiver brings his or her own set of cultural values into the caring interaction (Doswell & Erlen, 1998; Wros, Doutrich, & Izumi, 2004). In this regard, Konishi et al. (2009) writes that; although professional values is a global issue, culture gives them shape and emphasizes on differences. Every culture talks about individual ethics with different emphases and importance. From the other side, if we believe that values are derived from culture, we should assume that individual and professional values need clarification and should be emphasized specifically (Konishi et al., 2009). although knowledge and value in nursing have been developed for a long time and exclusively from West to other parts of the world, this phenomenon raised many questions about knowledge and awareness of professional values for non-western countries nurses, so that socialization is severely influenced by western culture, individual and clinical life related to the traditional culture of the person himself/herself (Davis, Tschudin, & De Raeve, 2006). However, perceiving some of the ethical concepts is hard and abstract for many nurses because of lack of evidence and examples for doing that practically. Nowadays, although boundaries in the world are removed rapidly, some values such as: “togetherness, harmony, politeness and respecting older people” are still there in the Japanese culture which caused moral distinction between Western countries and Japan (Konishi et al., 2009). Knowing the influence of culture on professional values development for nursing students and the effect of culturally values based on ethical decision-making by practicing nurses might be better recognized by examining the influence of the respective worldview on ethical decision-making and priority values. Findings of the studies regarding *the effect of learners’ individual characteristics* also showed that, gender plays an important role in developing professional value, especially in female students. In this regard, it can be said that; nowadays, many male students consider nursing as a job and not a profession; actually they look at it as higher chance for entering university, with easy acceptance conditions, diverse career options and probably as a job. It is while female students look at it as a care profession and altruism. In agreement with the findings of the study, Rognstad et al. (2004) stated that; even in the recent 30 years, despite the effect of education factors such as; some progresses in nursing technology, still it seems that young women select nursing as a profession because of paying attention to the value of altruism (Rognstad et al., 2004). Findings achieved from the study indicated that; different factors such as; education, kind of attitude and culture influence development of professional values and ethical decision makings of the nurses remarkably. These findings entirely indicate that; all the educational centers should use different educational approaches with the aim of making the students familiar with professional values according to unique characteristics. From the other side, recognition of the students’ characteristics help the instructors to have better understanding of the way of different students’ growth for being professional and evaluate and use educational strategy according to cultural differences for professional growth of the students. This study has some limitations such as shortage of research studies with different approaches in the area of professional values of nursing instructors and students. Limited quantitative and qualitative studies indicate the necessity of more attention and researches regarding professional values. A review of the articles showed that; in the area of professional values, most of the studies by doing quantitative and qualitative study only assessed students and instructors’ perspective and attitude. From the other side most of the articles only used a standardized tool which was designed by Schank and Weis (1994 and 1997). Researchers in the present study had conducted their review study by searching in the available databases with the aim of achieving the original and abstract of the articles. Considering this issue that professional values and their effective factors are formed by social and cultural contexts; it is recommended to the researchers to explain and perceive approaches of professional values and nurses and students’ experiences in this regard by conducting studies with phenomenological, grounded theory and mixed- methods approaches and also designing standardized tools based on the special culture of the society. This issue is going to be a great help in this area and also it is going to help nursing professional growth and professionalization process.

## 5. Conclusion

Professional values among the students will be developed through education and achieving experience, perspective and attitude and also some cultural and individual factors. This issue is influenced by nursing instructors’ perspectives to the high extent since they are in a permanent relationship with students. Paying attention to instructors and students’ perspective is very important which should be considered in the nursing profession. Therefore, providing educational and care environments based on ethical and professional values in nursing is essential and it can be done by conducting broad studies.
